# Early Expanded Polytetrafluoroethylene Sheet Removal Due to Postoperative Infection in Frontalis Suspension Surgery Preserves Eyelid Elevation and Curvature

**DOI:** 10.7759/cureus.103307

**Published:** 2026-02-09

**Authors:** Shinjiro Kono, Motohiro Kamei

**Affiliations:** 1 Department of Ophthalmology, Aichi Medical University Hospital, Nagakute, JPN

**Keywords:** case report, congenital ptosis, expanded polytetrafluoroethylene sheet, eyelid curvature, infection, oculoplastic surgery, suspension surgery

## Abstract

In frontalis muscle suspension surgery for treating congenital ptosis, the use of expanded polytetrafluoroethylene (ePTFE) sheets often yields stable postoperative outcomes; however, it also carries the risks of foreign body reactions and infection, with few reports describing the detailed course of treatment in such cases. A 15-year-old girl was referred for management of right congenital ptosis. Preoperative margin reflex distance-1 (MRD-1) measurements were 1.5 mm on the right and 4.5 mm on the left, with corresponding levator function of 5 mm and 14 mm on the right and left, respectively. Levator advancement was considered insufficient to correct the asymmetry; therefore, a frontalis muscle suspension using an ePTFE sheet was planned. After positioning the sheet and forming double eyelids, MRD-1 was confirmed to be symmetrical bilaterally (4.5 mm), the curvature was appropriate, and the incision was closed. At the two-week postoperative suture removal, a small portion of the ePTFE sheet was visible through a gap in the wound at the eyelid margin; therefore, it was trimmed, and wound closure was subsequently confirmed. Six weeks after surgery, the patient presented with fever, eyelid redness, and pain. Infection was noted at the eyelid margin wound site; therefore, the ePTFE sheet was removed, followed by wound irrigation. After prompt ePTFE sheet removal, adequate eyelid elevation and curvature were equivalent to those prior to sheet removal, making this protocol a viable treatment option for similar cases.

## Introduction

Frontalis muscle suspension surgery using expanded polytetrafluoroethylene (ePTFE) sheets for congenital ptosis yields postoperative results that are more stable than those obtained using other materials such as fascia lata or silicon rods with lower rates of recurrence and eyelid dysfunction; however, it carries the risks of foreign body reactions and infection [1-6].

Reports on postoperative infection rates vary, and although they have decreased in recent years, they still occur at a measurable rate [1,3,5,6]. Management involves antibiotic administration or implant removal; however, the approaches are evaluated case by case, and only a few reports have described the detailed course of treatment [1]. This is the first detailed case report of a 15-year-old female patient who developed early infection following a frontalis muscle suspension procedure using an ePTFE sheet for right congenital ptosis, achieving favorable functional and cosmetic outcomes after emergency sheet removal.

Written informed consent was obtained from the patient and her mother for publication of this case report and any accompanying images. The surgical procedure and pre- and post-operative examinations were performed by one of the authors (S.K.).

## Case presentation

**Figure 1:**
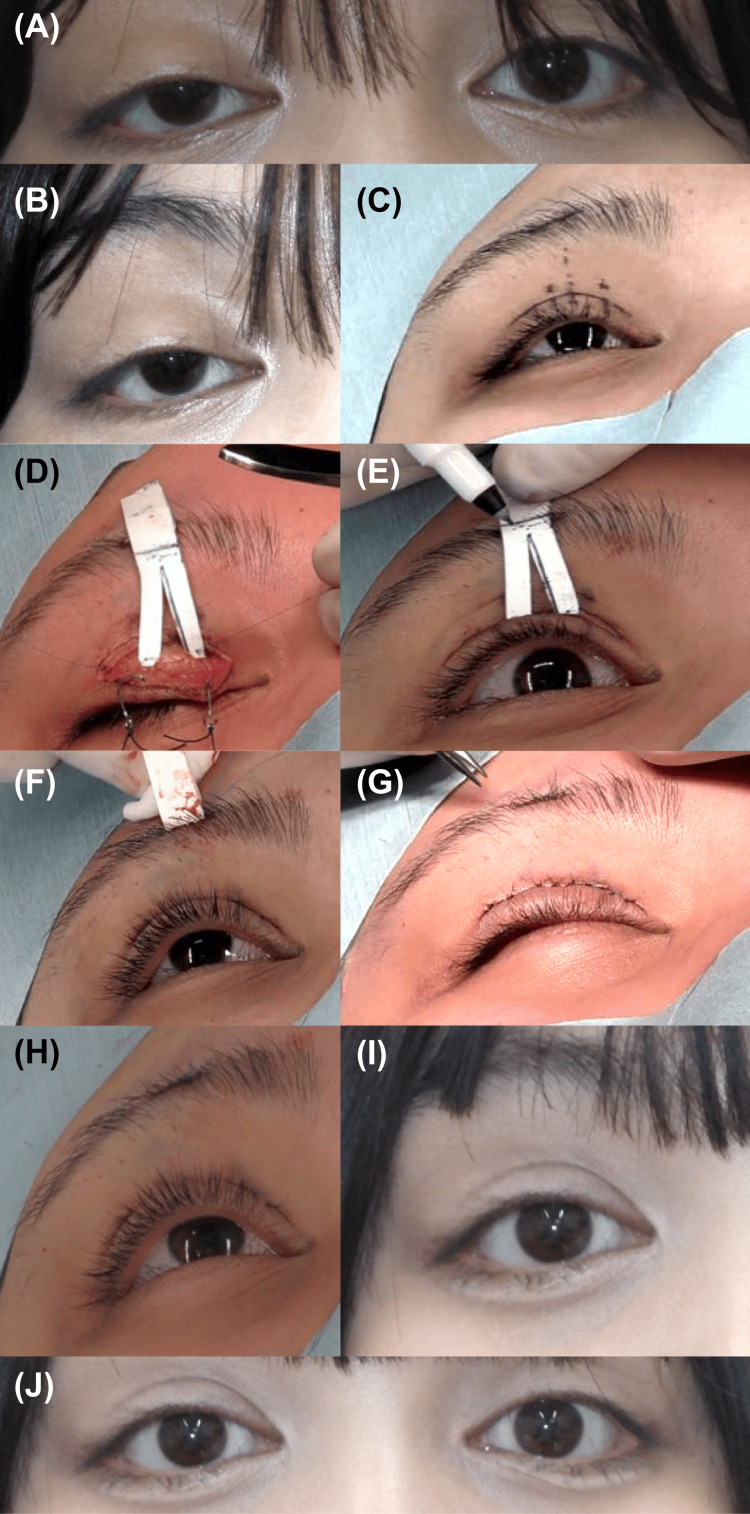
Initial surgery. (A) and (B) Preoperative facial photograph. Right congenital ptosis is noted. Margin reflex distance-1 (MRD-1) values are 1.5 mm and 4.5 mm on the right and on the left, respectively. (C) Designing the skin incision line and expanded polytetrafluoroethylene (ePTFE) sheet fixation position. (D) The sheet is sutured to the tarsal plate. (E) The sheet is manually elevated, and the degree of eyelid elevation is measured. (F) Forming double eyelids and fixation of the 18-mm portion from the lower edge of the sheet to the frontalis muscle. (G) and (H) Wound closure after trimming the excess portion of the sheet. (I) and (J) Image taken three weeks postoperatively. MRD-1 values are uniform at 4.5 mm bilaterally, and the eyelid curvature is symmetrical. Wound closure is complete. Images used with permission obtained from the patient and her mother.

In October 2024, a 15-year-old girl was referred for treatment of right congenital ptosis. The patient had no history of eyelid surgery and no known systemic disorders. Preoperative margin reflex distance-1 (MRD-1) measurements were 1.5 mm on the right and 4.5 mm on the left, with levator function of 5 mm and 14 mm on the right and left, respectively (Figure 1A, 1B). Levator advancement was considered insufficient to correct the asymmetry; therefore, a frontalis muscle suspension procedure was planned. A bifurcated ePTFE sheet (thickness 0.3 mm, vertical length: 45 mm, width: 7 mm; bifurcated portion length: 17 mm) was prepared. After designing the skin incision line and ePTFE sheet fixation position, both legs were sutured to the tarsal plate above the corneal limbus on the medial and lateral sides (Figure 1C, 1D). After positioning the sheet and forming double eyelids, the sheet was sutured to the frontalis muscle, 18 mm from the lower end. (Figure 1E, 1F). An excess portion of the sheet was trimmed. MRD-1 was confirmed to be symmetrical bilaterally (4.5 mm), the curvature was appropriate, and the incision was closed (Figure 1G, 1H)

**Figure 2:**
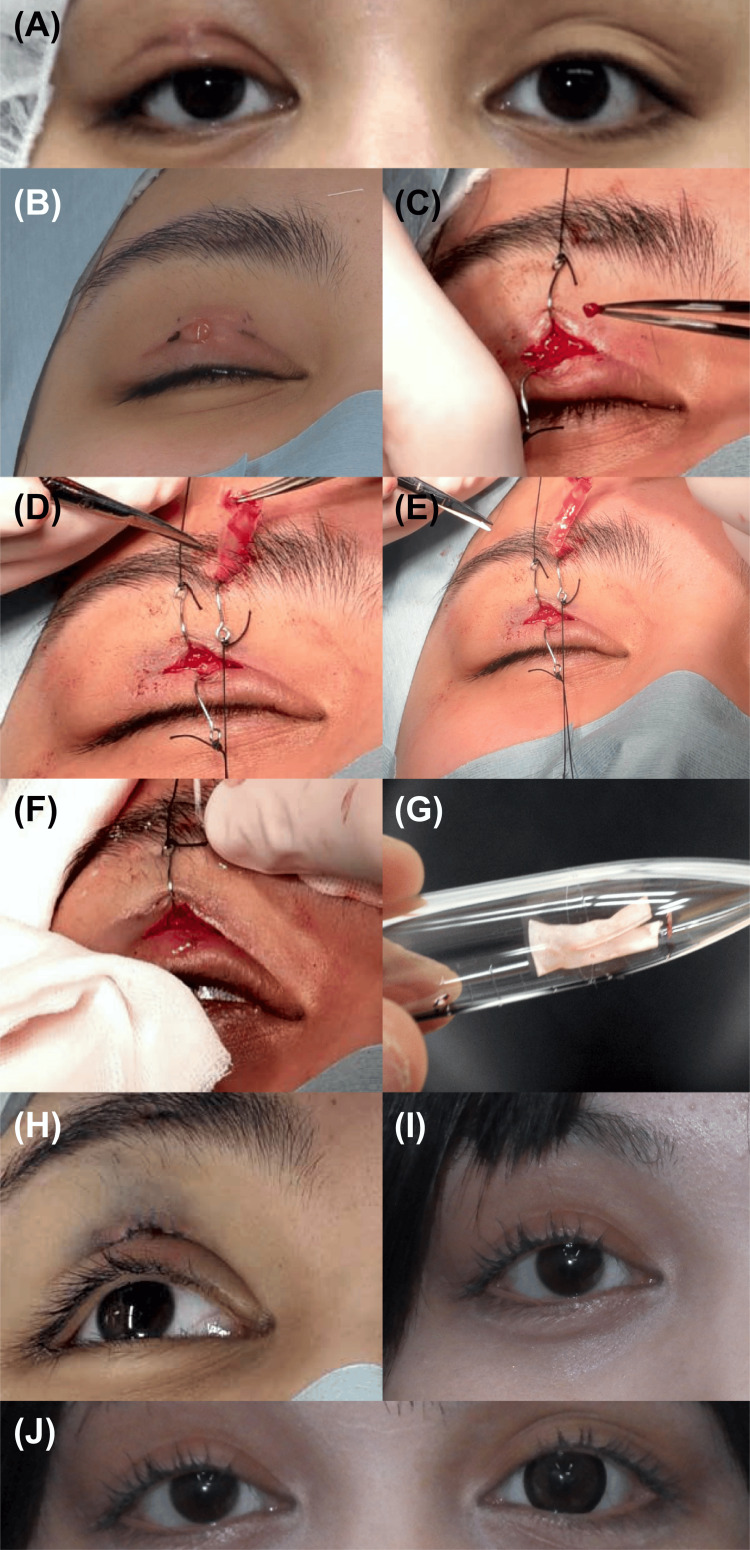
Sheet excision surgery. (A) and (B): Preoperative view of sheet removal surgery (six weeks after initial surgery). Tenderness and erythema due to infection are noted at the eyelid margin. Yellowish purulent discharge is observed from the wound site at the eyelid margin. Preoperative Margin Reflex Distance-1 (MRD-1) value on the right is 3.5 mm due to swelling. (C): Granulation tissue emerges upon incision. (D) and (E): Sheet removal after detaching tarsal plate and frontalis muscle sutures. (F): Wound irrigation with saline. (G): Extracted ePTFE sheet. Culture tests on the expanded polytetrafluoroethylene (ePTFE) sheet are negative, and no skin flora, Pseudomonas aeruginosa, or methicillin-resistant Staphylococcus aureus (MRSA) is detected. (H): After wound closure. Sufficient eyelid elevation with appropriate curvature. MRD-1 value on the right improves to 4.5 mm. (I) and (J): Three months after the second surgery: eyelid margin redness and pain have subsided, and MRD-1 values are uniform at 4.5 mm bilaterally. The right eye shows no abnormality. The left eye wears a colored contact lens. Images used with permission obtained from the patient and her mother.

At the 2-week postoperative suture removal, a small portion of the ePTFE sheet was visible through a gap in the wound at the eyelid margin; the exposed segment was trimmed. Three weeks postoperatively, MRD-1 values were uniform at 4.5 mm bilaterally, and the wound had healed(Figure 1I, 1J). Follow-up observation was continued. Six weeks after surgery, the patient reported fever, eyelid redness, and pain; therefore, an emergency visit was requested (Figure 2A). Infection was noted at the eyelid margin wound site, and the ePTFE sheet was immediately removed without the course of antibiotic administration, followed by wound irrigation (Figure 2B-2F). Culture tests on the ePTFE sheet were negative, and no skin flora, Pseudomonas aeruginosa, or methicillin-resistant Staphylococcus aureus (MRSA) were detected (Figure 2G). After ePTFE sheet removal, adequate eyelid elevation and curvature were maintained (Figure 2H). Following surgery, the patient took cefcapenpivoxil orally for three days as usual, followed by an additional six days of oral levofloxacin. At the time of suture removal, no signs of infection were observed, so oral medication was discontinued. Three months after the second surgery, the eyelid pain and redness subsided, and the patient's course was favorable (Figure 2I, 2J).

## Discussion

We report the case of a 15-year-old female who maintained favorable surgical outcomes following early detection and prompt removal of an ePTFE sheet due to postoperative infection.

This case involved an early infection originating from the wound site at the eyelid margin, which was confined to the eyelid margin at the time of examination. The incidence and severity of postoperative infections vary and have been reported to occur within 1 month or over 3 months after surgery [5]. Some cases can be managed with antibiotics alone or by removing the sheet, whereas others may require reoperation because of recurrent ptosis after sheet removal [1,3,5,6]. The patient was anxious because of the resulting swelling and pain; to prevent prolonged infection and avoid leaving visible sequelae, we decided to remove the ePTFE sheet immediately without first observing the course of antibiotic administration. As a result of prompt removal, contamination of the excised ePTFE sheet was limited to the lower edge only, and culture results were negative.

The intraoperative findings revealed sufficient fibrovascular tissue formation along the sheet, then, after the sheet removal, eyelid elevation and an appropriate curve were maintained. A previous study indicated that fibrovascular tissue formation along ePTFE sheets typically occurs within 14-28 days of implantation in humans [2,7]. Animal studies have reported that fibrovascular tissue covers ePTFE at the 5-week post-implantation time point [8]. In this case, sufficient fibrovascular tissue formation along the ePTFE sheet occurred 6 weeks after surgery, likely enabling maintenance of eyelid elevation and curvature after the sheet removal. Prolonged infection may destroy the proper fibrovascular ingrowth structure, potentially preventing sustained eyelid elevation after ePTFE sheet removal. Therefore, in cases where infection is detected early and the fibrovascular tissue is sufficiently developed, prompt sheet removal is advisable.

Thorough attention to wound closure is essential to prevent infection [1]. As the eyelid is subjected to frequent external stimuli, the ePTFE sheet must be securely positioned. In this case, the meticulous double eyelid creation in a young female patient may have resulted in slightly inadequate wound closure. To ensure secure closure, the ePTFE sheet should be positioned without overlapping the double-eyelid crease line.

## Conclusions

To conclude, in cases where fibrovascular tissue is sufficiently developed in early-stage infections following ePTFE insertion for congenital ptosis surgery, prompt sheet removal can maintain eyelid elevation and an appropriate curve, making it a viable treatment option. Further studies or case series are needed to confirm the generalizability of this management strategy.
